# Carbon Materials Prepared from Invading Pelagic Sargassum for Supercapacitors’ Electrodes

**DOI:** 10.3390/molecules28155882

**Published:** 2023-08-04

**Authors:** Sandra Roche, Christelle Yacou, Corine Jean Marius, Ronald Ranguin, Marckens Francoeur, Pierre-Louis Taberna, Nady Passe-Coutrin, Sarra Gaspard

**Affiliations:** 1 Laboratory «Connaissance et Valorisation: Chimie des Matériaux, Environnement, Énergie» (COVACHIM–M2E–EA 3592), Faculté des Sciences Exactes et Naturelles, Université des Antilles, B.P. 250, CEDEX, 97157 Pointe-à-Pitre, France; 2 CIRIMAT, UMR CNRS 5085, Université Paul Sabatier Toulouse III, 118 Route de Narbonne, 31062 Toulouse, France; 3 RS2E, Réseau Français sur le Stockage Électrochimique de l’Énergie, FR CNRS 3459, CEDEX, 80039 Amiens, France

**Keywords:** activated carbon, sargassum, supercapacitor, energy storage, biomass

## Abstract

Since 2011, substantial amounts of pelagic Sargassum algae have washed up along the Caribbean beaches and the Gulf of Mexico, leading to negative impacts on the economy and the environment of those areas. Hence, it is now crucial to develop strategies to mitigate this problem while valorizing such invasive biomass. This work deals with the successful exploitation of this pelagic Sargassum seaweed for the fabrication of carbon materials that can be used as electrodes for supercapacitors. Pelagic Sargassum precursors were simply pyrolyzed at temperatures varying from 600 to 900 °C. The resultant carbonaceous materials were then extensively characterized using different techniques, such as nitrogen adsorption for textural characterization, as well as X-ray photoelectron (XPS), Fourier transform infrared spectroscopies (FT-IR) and scanning electron microscopy (SEM), to understand their structures and functionalities. The electrochemical properties of the carbon materials were also tested for their performance as supercapacitors using cyclic voltammetry (CV), the galvanostatic method and electrochemical impedance spectroscopy analyses (EIS). We managed to have a large specific surface, i.e., 1664 m^2^ g^−1^ for biochar prepared at 800 °C (CS800). Eventually, CS800 turned out to exhibit the highest capacitance (96 F g^−1^) over the four samples, along with the highest specific surface (1664 m^2^ g^−1^), with specific resistance of about 0.07 Ω g ^−1^.

## 1. Introduction

**Figure 1:**
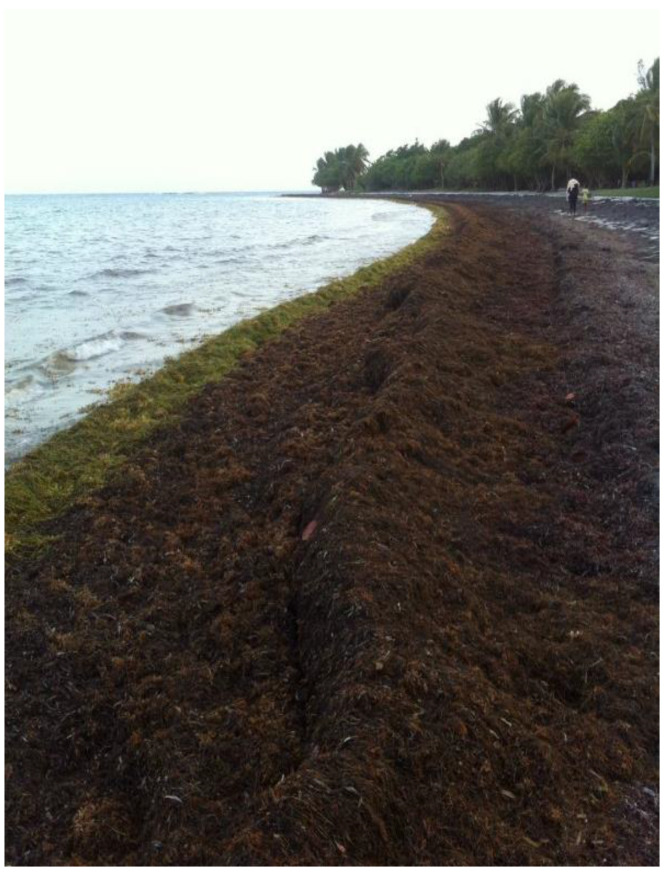
Picture of floating algae, including pelagic Sargassum, arriving on the coast at Saint-François in Guadeloupe.

The Caribbean area and the Gulf of Mexico are currently facing major crisis events of pelagic *sargassum* spp. seaweeds that are washing ashore along beaches in massive amounts (Figure 1). Such invasions were observed for the first time in 2011, followed by more or less serious episodes [1,2]. When algae wash up on beaches, the coastal ecosystems, the natural environment and the habitants’ lifestyles can be quickly altered. In addition, the economy of those areas is dramatically threatened, including the tourism and the fishing activity that is impacted due to reduced access to the sea. 

Therefore, valorizing such a huge amount of biomass is currently strongly considered for several applications. *Sargassum* spp. have been studied as precursors of carbon materials, which were applied for the removal of pollutants and heavy metals in water [2,3,4,5,6].

Herein, we propose an original pathway towards the valorization of such invasive *Sargassum* spp. through the development of valuable and low-cost carbon materials for energy storage applications, followed by the investigation of their structural, physico-chemical and electrochemical characteristics. Indeed, it is known that seaweeds can serve as suitable precursors for the fabrication of many carbon-based materials, including activated carbon fibers or electrodes [7,8]. Depending on the chemical composition and pyrolysis conditions of the raw material, high specific-energy material for electrochemical double layers capacitors (EDLC) can be obtained [9]. EDLC, also called supercapacitors, are devices exhibiting specific energy and power between batteries and dielectric capacitors. Thanks to their time constant (a few seconds), they are suitable for energy harvesting such as energy break recovery (electric cars). Nevertheless, to extend their utilization, the increase in their specific energy together with the decrease in their operating and synthesis cost remain the most pressuring challenges. 

Original and cheap electrodes derived from highly available Sargassum seaweeds from the Caribbean area were developed and further investigated for their electrochemical characteristics as supercapacitors. A very simple route was chosen to synthetize the materials consisting of a one-step pyrolysis of the raw seaweed at different temperatures. The developed materials were then characterized using the thermogravimetric technique (TG), scanning electron microscopy (SEM), Fourier transform infrared (FT-IR) spectroscopy and X-ray photoelectron spectroscopy (XPS) to better understand their structures and functionalities.

## 2. Results and Discussion

### 2.1. Materials’ Characterization

**Figure 2:**
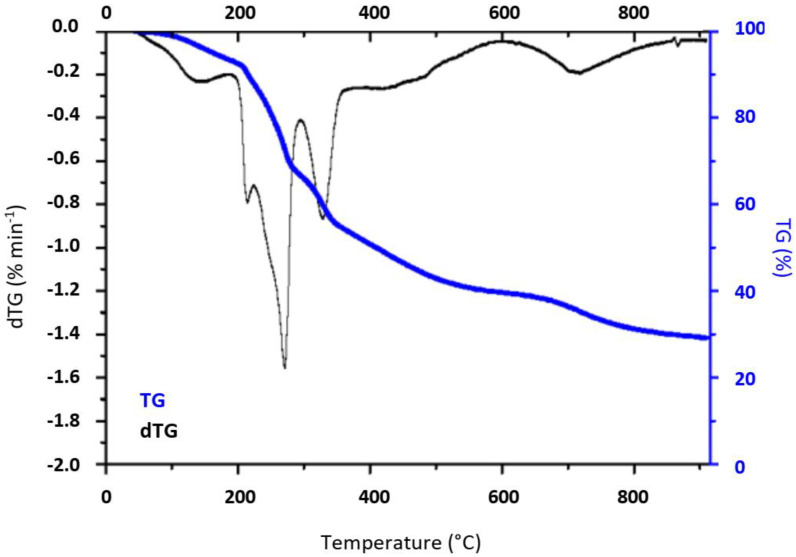
Thermogravimetric analysis profiles of as-received Sargassum algae (S_raw_).

In general, thermal treatment of brown algae consists of a stepwise pathway corresponding to the decomposition of several biopolymers including polysaccharides (alginic acid, sulfated fucans) and minerals [10]. As such, thermogravimetric (TG) analysis is a good method to identify biochemical content of the native Sargassum (S_raw_) while simulating pyrolysis. As shown in Figure 2, the mass loss of S_raw_ occurs in three temperature zones: below 180 °C, between 200 and 450 °C, and above 500 °C, indicating a stepwise pathway of the decomposition of the biopolymers aforementioned. The dehydration of the sample (moisture in both the cellular envelope and surface of alga) was firstly observed at a temperature below 180 °C. The devolatilization step occurred in the second stage with a main degradation of the carbohydrates between 200 and 300 °C, followed by the proteins’ weight loss occurring between 300 and 400 °C [11]. Upon pyrolysis, a slight and gradual weight loss was observed, probably due to further devolatilization of the formed carbon in tandem with little volatile metal loss. Nevertheless, above 600 °C, no significant weight loss was observed, indicating thermal stability of the char from this temperature. A final zone of mass loss is noted between approximately 650 and 800 ° C. The material seems to stabilize after this area at 900 °C. Consequently, the starting temperature chosen to pyrolyze the precursor is 600 °C, giving rise to the CS600 sample, followed by samples CS700, CS800, CS900 resulting from pyrolysis at 700, 800 and 900 °C respectively.

**Figure 3:**
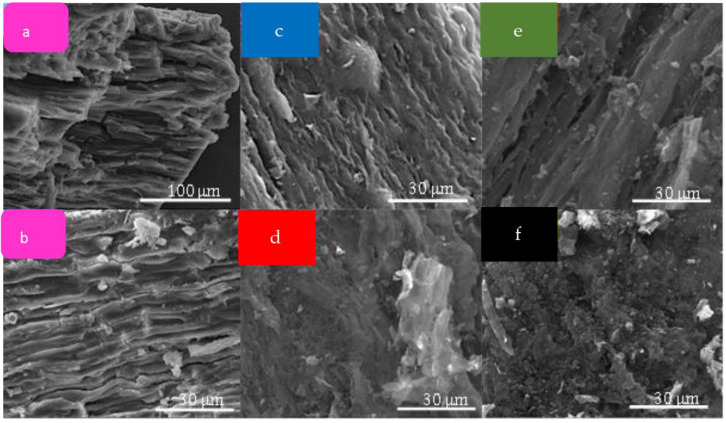
SEM micrographs of (**a**,**b**) raw Sargassum, (**c**) CS600, (**d**) CS700, (**e**) CS800 and (**f**) CS900.

**Figure 4:**
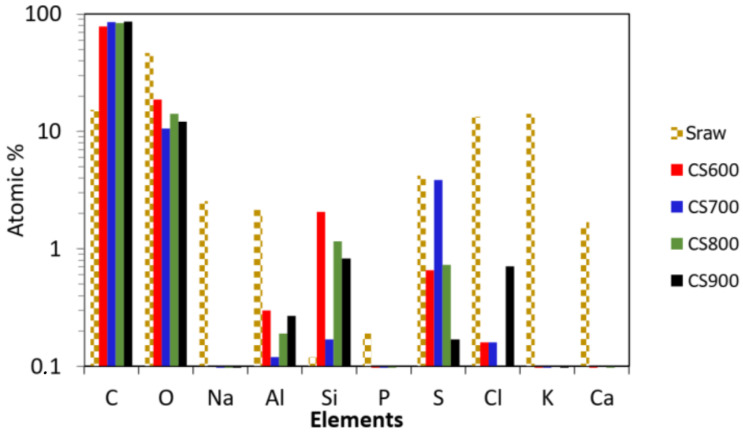
Semi-quantitative approximation of an elemental analysis by EDX of raw Sargassum and its derived carbons CS600, CS700, CS800 and CS900 (EDX analysis).

Scanning electron microscopy (SEM) was carried out to evaluate the morphology of materials before and after pyrolysis. As shown in Figure 3, raw Sargassum (S_raw_) exhibits a dense and stratified microstructure in line with previous reported SEM micrographs of Sargassum and other brown algae [12,13]. This structure mainly contains the cell wall of the seaweed constituted of polysaccharides (alginic acid and sulfated fucans), proteins and a little of cellulose [14]. After thermal treatment at high temperature, SEM images reveal slightly damaged surfaces probably due to the release of volatile compounds during the carbonization process. However, no obvious macropores could be detected in the carbons, indicating a limited macroporosity obtained after pyrolysis. Bulk elemental compositions shown in Figure 4 indicate traces of inorganic maters in the raw algae, including light metals S, K, Al, Na, Cl and Ca. Besides the organic compounds cited above, salt, oxides or hydroxides (originally dissolved or suspended in the sea water) can be precipitated or adsorbed on the biomass surface [15]. After thermal treatment at different temperatures, only sulfur was detectable (probably under the form of sulfate or sulfonate), and the other elements were present only at trace level (i.e., the sum of these elements is less than 1.0 at.%). It can be assumed that the element sulfur, which was blocked in the matrix of the algae, is released with the increase in temperature.

**Figure 5:**
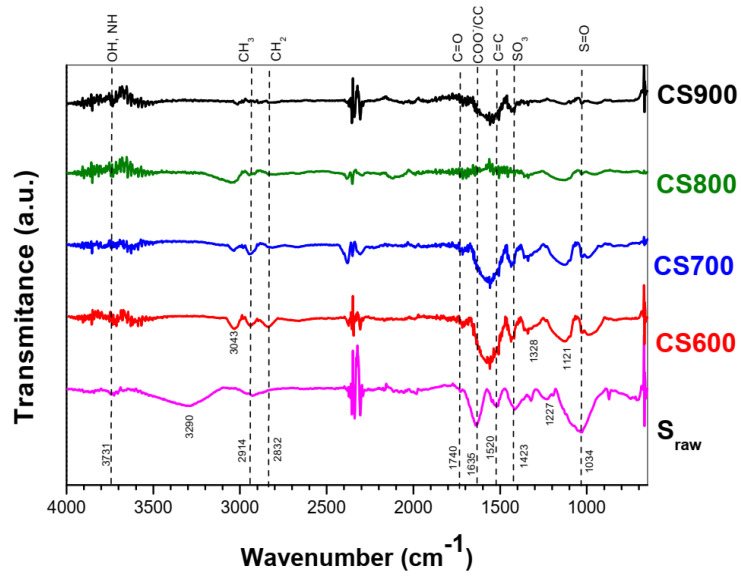
FT-IR spectra of raw Sargassum algae and its derived chars CS600, CS700, CS800 and CS900.

The evolution of the chemical structure of Sargassum seaweed (S_raw_), consequent to the pyrolysis at different temperatures, was analyzed via FT-IR spectroscopy. Table 1 and Figure 5 present the results of the FT-IR analysis and the peak assignment. Firstly, the S_raw_ spectrum shown in Figure 5 exhibits a broad vibration band in the range of 3000–3600 cm^−1^ besides peaks at 3040 cm^−1^, 2920 cm^−1^, 1630 cm^−1^, 1416 cm^−1^, 1230 cm^−1^ and a strong band at 1020 cm^−1^. The assignment of those bands can be related to the main functional groups usually found in the cellular envelope of Sargassum, such as alcohols (C–O–H) and amides (N–H), hydrocarbons (C–H), carboxyl acid/carboxylate (COOH/COO^−^), sulfonate (–SO_3_) and ethers (C–O–C) [13,16]. When analyzing FT-IR spectra of CS600 to CS900, the intensity of several peaks decreased or disappeared relative to the S_raw_ spectrum, suggesting a successful transformation of the biomass into graphitic structure via the decomposition of carbon groups and subsequent release of their by-products as volatile matter. The aromatization character of these samples was evidenced by the shift to lower frequencies of C=O bands (1630 cm^−1^) to form aromatic C=C bonds at 1570 cm^−1^ [17,18]. Another noteworthy observation is the significant decrease in the amount of oxygenated groups including C–O–C and O–H, which further agrees with the polyaromatic character of those samples [19].

**Figure 6:**
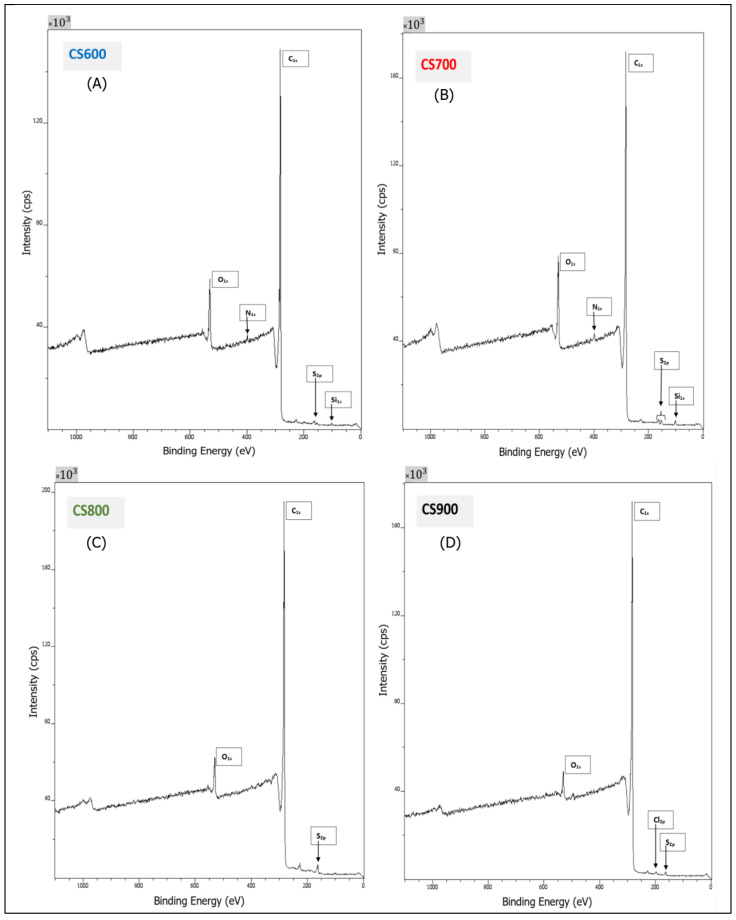
XPS spectrum analysis of Sargassum algae derived chars CS600 (**A**), CS700 (**B**), CS800 (**C**) and CS900 (**D**), and assignment peaks.

The surface chemistry of Sargassum carbons was further investigated by XPS to corroborate the results of both EDX analysis and FT-IR spectroscopy. Figure 6 and Table 2 resume the results of assignments and percentage of different elements detected by peaks: O_1s_ (532.9–533.1 eV), C_1s_ 284.6–284.65 eV), Si_2p_ (103.55–103.6 eV), N_1s_ (401.05 eV), S_2p_ (163.85–163.9 eV), Cl_2p_ (199.6 eV). 

**Figure 7:**
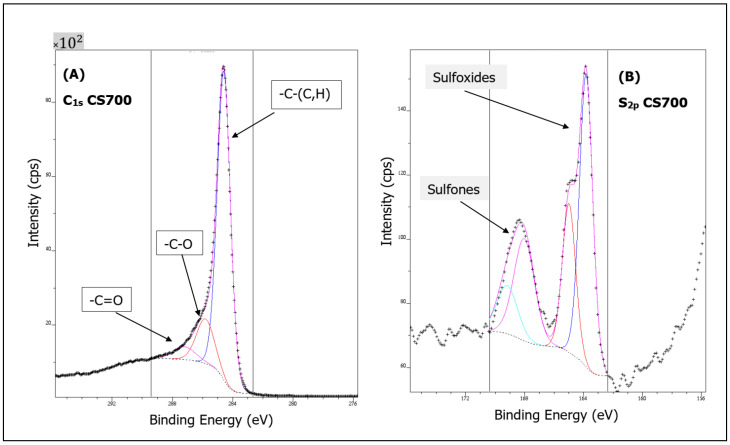
Deconvoluted C_1S_ (**A**) and S_2p_ (**B**) XPS spectrum for CS700 sample.

As expected, C and O were detected in all carbon samples and the O/C ratio indicating the degree of surface oxidation was calculated (Table 2). It can be observed that beyond a heat treatment at 700 °C, this ratio decreases significantly, suggesting that oxygen is being removed during pyrolysis, whilst the percentage of carbon increases. Likewise, this correlates well with the evolution of the chemical structure of the samples during pyrolysis observed via FT-IR. Graphitization was confirmed while the increase in graphite type was in tandem with temperature, from 79.6% at 600 °C to 82.4% at 900 °C. As shown in Figure 7, the deconvolution of the C1_s_ spectrum of CS700 and of all of them (CS600, CS800 and CS900 not shown) consists of several peaks: (I) graphite type (284.1–284.4 eV), (II) carbonyl groups (285.5–286.1 eV), (III) carboxyl and ester groups (286.3–287.6 eV). The carbonyl groups of C_1s_ link to O-C in the O_1s_ spectrum usually located between 530.7 and 531.2 eV and carboxyl/ester groups of C_1s_ link to O=C, OH in the O_1s_ spectrum usually located between 531.8 and 532.7 eV. Analyzing those data further provides evidence about the fact that the content of graphitic carbon increases as a function of the temperature, while the amount of oxygenated groups decreases as observed by FT-IR spectroscopy. On closer inspection, sulfur was also detected in samples CS700, CS800 and CS900, while silicon was measured for CS700 and CS900, which is consistent with the EDX analysis. Two types of sulfur species on the surface of the CS700, CS800 and CS900 could be determined, thiophenic and oxidized sulfur species (sulfoxide and sulfone), with corresponding peaks at 163.8–164.9 eV and 168.03–199.19 eV, respectively (Figure 7). Silicon was detected in samples prepared at the lowest pyrolysis temperature, CS600 and CS700, and chlorine was detected in CS900 alone. Note that the percentage of sulfur decreases as the pyrolysis temperature increases.

**Figure 8:**
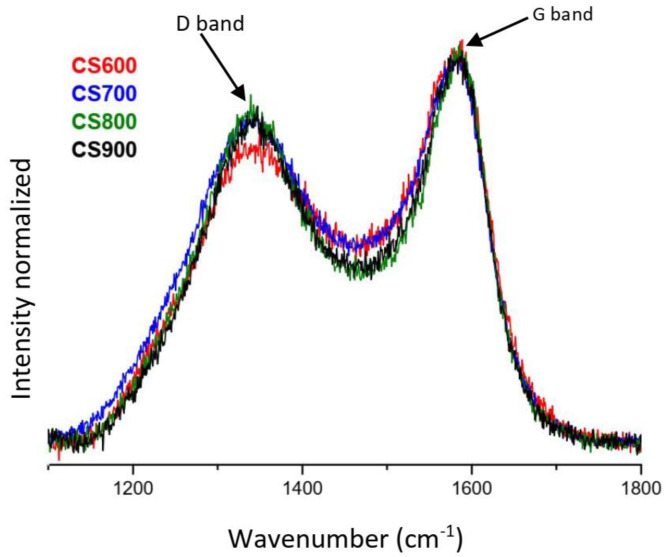
Experimental Raman spectra normalized of CS samples.

It is generally accepted that the evolution of coal structure is complex [20]. As representative measures of graphitic structures, the spectrum peaks at 1350 cm^−1^ and 1590 cm^−1^ are known as D band and G band, respectively [20,21,22]. D band is caused by disordered structure or in-plane defects between the basic structural units, whereas G band arises from the breathing of the aromatic ring for amorphous carbonaceous materials [20]. Figure 8 shows the Experimental Raman spectrum normalized on the G band. A focus on D band and G band only is not the best analyzer to characterize the complex structure of carbon because we lose some information [20]. The G bands are perfectly identical for all samples. Except for CS600, all of the D bands are identical position. We note a decrease in the inter-band between D and G. Clearly, disorder decreases with the increase in temperature. These results are in correlation with the XPS results. Indeed, there is graphitic carbon (C2p at 0.32%) in CS900. 

**Figure 9:**
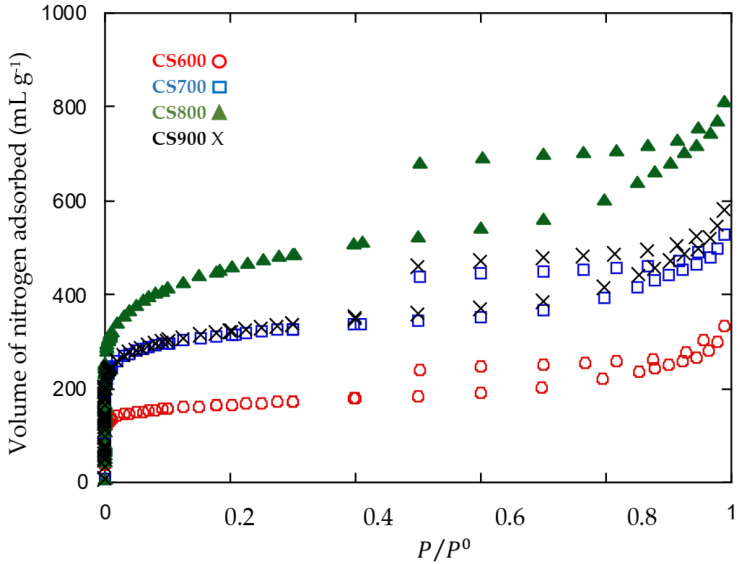
Nitrogen adsorption–desorption isotherms of CS samples.

Nitrogen adsorption and desorption isotherms at 77 K are shown in Figure 9. All samples exhibit typically type IV isotherms with hysteresis loops at a relative pressure higher than 0.4, which suggests a well-developed mesoporosity. Therefore, CS carbons can be classified as mesoporous chars with significant volume of micropores ranging from 26 to 43% of porous volume (Table 3). High BET surface area values were found, starting from 621 m^2^ g^−1^ for CS600 to 1664 m^2^ g^−1^ for CS800. Similarly, to the results found for another tropical alga *Turbinaria turbinata*, the pyrolysis temperature of 800 °C gives the highest specific surface area, and optimum micropore and mesopore volumes [8]. Increasing the temperature to 900 °C leads to a loss of surface area, which is probably due to pore-wall breakage.

### 2.2. Electrochemical Characterization

#### 2.2.1. Voltammetry

**Figure 10:**
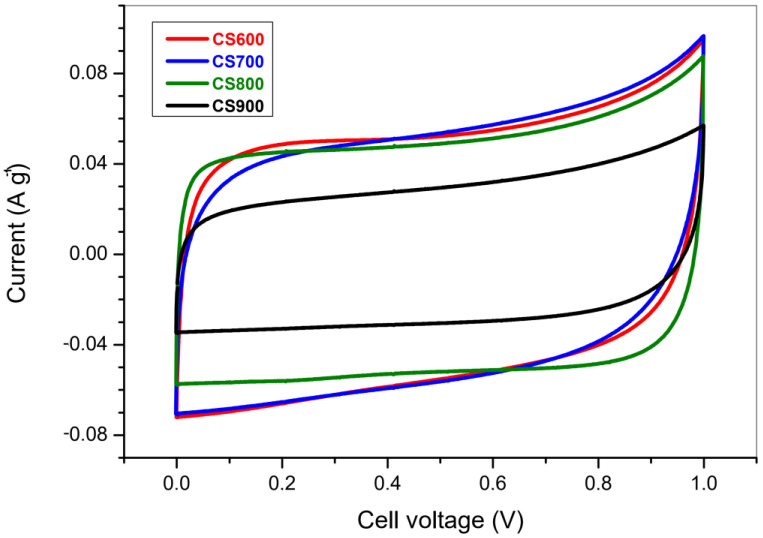
Voltammetry second cycle curve obtained for CS600, CS700, CS800 and CS900. Electrical current is the answer for an imposed cell voltage varying from 0 to 1 V in 1 M H_2_SO_4_.

Figure 10 displays the cyclic voltamograms (CVs) obtained at 1 mV s^−1^ for the CS carbons in 1 M H_2_SO_4_. Analysis of these measurements reveals a decent capacitive behavior. The cycles have no peaks, which means no clear faradic reactions in the voltage domain of 0 to 1 V. It can also be noticed that the CVs are less sloping (leakage of current) while increasing the thermal treatment temperature. It can be ascribed to the aforementioned decrease in the amount of oxygen functional groups at a higher temperature. A rise of the current is observed beyond 0.8 V, which is consistent with the electrochemical stability of such electrolyte. 

#### 2.2.2. Galvanostatic Charge-Discharge

**Figure 11:**
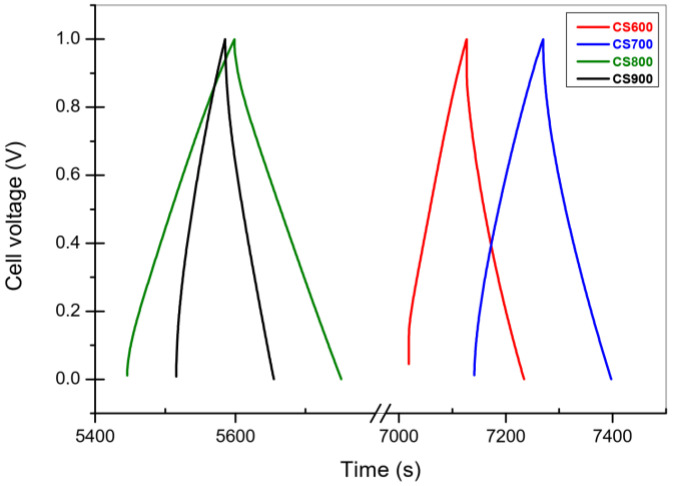
Galvanostatic cycles for sample CS800. Current value was fixed at 270 mA g^−1^ and the voltage varied as function of the time for successive charges and discharges.

To refine the capacitance value previously obtained, galvanostatic measurements were performed for the CS carbons in 1 M H_2_SO_4_ (Figure 11). For each experiment, the potential varied from 0 to 1 V and current density was fixed at 4 mA cm^−2^. Figure 11 shows that CS800’s discharge has a low slope compared to the others; this implies the biggest capacitance. The active material capacitance (96 F g^−1^ for CS800) was determined from the discharge slope of the last cycle, with cyclability testsperformed. Similarly, to our previous work published on carbons derived from the brown algae *Turbinaria turbinata*, the capacitance value is maximal for carbons pyrolyzed at 800 °C under the same conditions, in agreement with voltammetry results reported by Pintor et al. [8]. It is believed that the high BET surface area (1664 m^2^ g^−1^) in tandem with the large microporous volume (0.92 cm^3^ g^−1^) for CS800 char may be responsible for this optimum capacitance value. It is also noteworthy to specify that the size of the pore allows the de-solvated ion to enter a micropore after a rapid diffusion through the mesopores [23].

**Figure 12:**
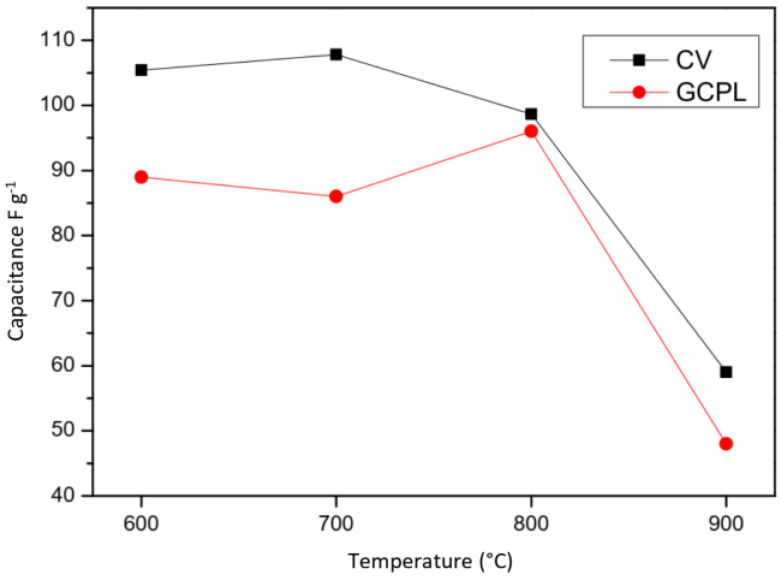
Capacitance values versus temperature obtained by CV (black) and by GCPL (red).

Figure 12 resumes the capacitance calculus obtained by CV and GCPL test. It confirms the same behavior with the temperature after 800 °C the capacitance decrease. However interestingly, among all tested carbons, CS800 exhibits the best behavior with a quasi-constant current of 0.05 A g^−1^. 

The preliminary test obtained by CV already indicated a trend of the capacitance, which decreases after 800 °C. Since capacity measurements using the GCPL are more precise, we will consider these for the rest of the discussion.

#### 2.2.3. Electrochemical Impedance Spectroscopy

**Figure 13:**
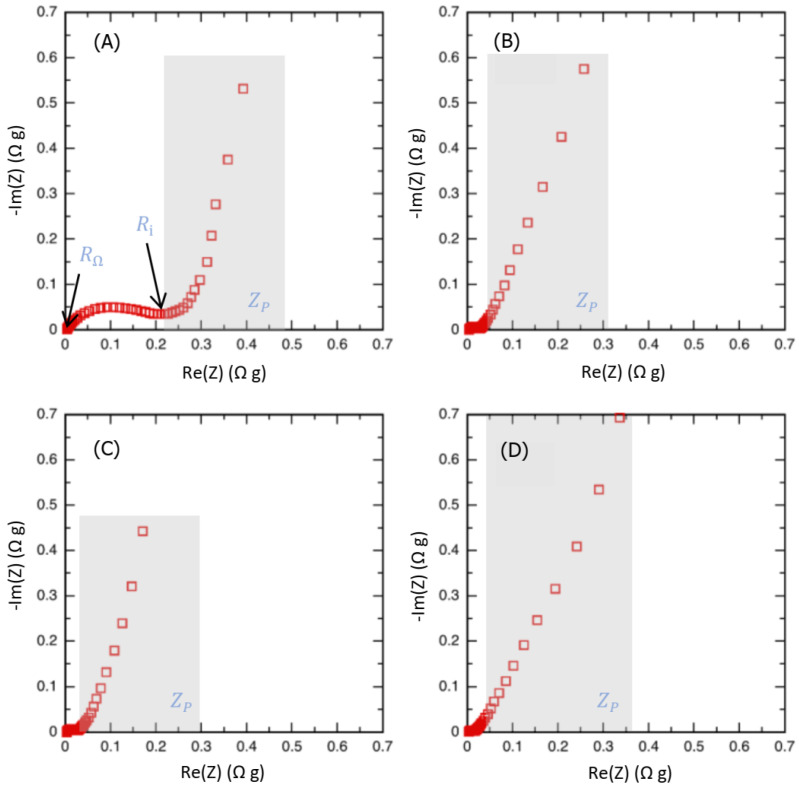
Electrochemical impedance spectroscopy curves for CS600 (**A**), CS 700 (**B**), CS 800 (**C**) and CS900 (**D**) samples. Curve represents the Nyquist diagram giving opposite imaginary part -Im(Z), versus real part Re(Z), of the complex impedance.

Another crucial electrochemical parameter of the CS samples, i.e., their resistance, was investigated via electrochemical impedance spectroscopy (EIS). The shape of the impedance diagram (i.e., Nyquist plot) indicates a capacitive behavior for all the samples (Figure 13). Three distinct domains were observed in the Nyquist diagram: a high frequency loop, usually linked to the current collector/active material interface, giving the *R_i_* resistance [24] but that could be related to poor electronic conductivity within activated carbon grains. Our results suggest the latter case is more likely to occur since the current collector used was an aluminium foil in all cases. As observed, this high frequency loop seems to depend on the carbon thermal treatment temperature. Then at lower frequency, a Warburg-like region and a quasi-vertical line show up. The high frequency intercept is dominated by the ionic resistance of the bulk electrolyte (*R*_Ω_) entrapped in the separator between the two electrodes, whereas the Warburg-like region comes from the ion motion inside the pores (*Z_P_*). The modeling of the Warbur-like and vertical region are usually obtained using a transmission line model [25]; the impedance of this transmission line can be calculated in Equation (1)

(1) $$Z_{p}\left(\omega \right)=\frac{R_{p}}{{\left(j\omega \tau \right)}^{\frac{\alpha }{2}}cotanh\left[{\left(j\omega \tau \right)}^{\frac{\alpha }{2}}\right]}$$

A vertical line (low frequency) is obtained when the total porosity is accessed by leading the full capacitance of the cell (C_SC_). The *Z_P_* impedance accounts for the R-C network coming from the pore size distribution leading to different access time to the pores [26]. *Z_P_* (in Ω g) is characterized by a time constant *τ* for the determination of its in-pore equivalent specific resistance *R_P_* (Ω g^−1^) and in-pore specific capacitance *C_P_* (F g^−1^) [26]. A good estimate of the effective in-pore ionic resistance is then *R_P_*/3. Moreover, the equivalent series resistance (ESR) obtained from the sum of the three values *R*_Ω_, *R_i_* and *R_p_*/3, and the equivalent cell capacitance (C_SC_) calculated mainly from the low frequency range related to the supercapacitor behavior can also be reported [8,24]. Table 4 depicted the values obtained for each CS sample.

Overall, it is noted that the CS600 sample is the one with the highest resistance values, while the CS800 sample has the lowest resistances. On the other hand, apart from the CS900 sample, all samples have capacities greater than 85 F g^−1^. The R in bulk electrolyte resistance indeed does not have a great difference of order of magnitude; 0.0035 Ω g ≤ R_Ω_ ≤ 0.0048 Ω g, with CS600 being the sample with the highest value. Looking at the effective in-pore ionic resistances, we note that all values are low ($R_{P}\;$≤ 0.016). This is suggesting a good ability for the ion to move through the porous structure. Moreover, it also can be seen that the $R_{i}$ values of CS600 and CS900 (0.21 and 0.12 Ω g) are higher than those of the other activated carbons, meaning both a poorer electron transport and an ion transport occur within the activated carbon [27]. 

The smallest equivalent series resistance (ESR) values are obtained for CS800 (0.073 Ω g) and CS700 (0.085 Ω g) due to the lower effectiveness in pore resistance (0.016 Ω g) and low carbon electronic conductivity for both samples—*Ri* values of 0.053 and 0.065 Ω g are obtained for CS800 and CS700, respectively (Table 4). The larger ESR value obtained for the CS600 sample (0.33 Ω g) compared with that of the other CS sample is consistent with the smallest total porous volume, 0.36 cm^3^ g^−1^, its higher oxygen group content and its higher disorder degree. Indeed, a larger pore volume, a lower oxygen group content and a higher carbon order are leading to higher capacitances and a lower series resistance for the carbons made in this study.

Finally, the equivalent series resistance (ESR) obtained from the sum of the three previous values allows to conclude on the overall behavior via the PEIS analysis of the samples. Although having a specific capacity of 89 F g^−1^, the sample CS600 remains the one with the highest ESR (0.33 Ω g^−1^). That is, CS800 can be considered as the best of the four samples with the highest specific capacitance (96 F g^−1^) and the lowest equivalent series resistance of 0.073 Ω g^−1^.

Table 5 gives capacitance values, extracted from the literature, obtained for activated carbons and biochars prepared from different precursors, using H_2_SO_4_ as electrolyte at 0.5 M or 1 M. Capacitance values around 32 to 302 F g^−1^ were found. The highest capacitance values are generally obtained for samples prepared by KOH activation. It should be noted that biochars obtained from a simple pyrolysis at 600 °C of a brown algae, *Lessonia Nigrescens*, have an exceptional high capacitance value of 264 F g^−1^. One interesting thing is that by considering the CS600 and CS900 with LN600 [7] and LN900 [7] samples, it is observed that they have the same behavior with regard to the evolution of the S_BET_ during the increase in temperature. The results obtained in this work for Sargassum biochar are in the range of those obtained for other pyrolyzed biomass. When considering the recurring events of Sargassum washing ashore, which rends this source of carbon easily available, the production of biochars by a simple pyrolysis step of this biomass could be of great interest for their possible application in electrochemical supercapacitors.

## 3. Materials and Methods

### 3.1. Carbon Materials’ Preparation

Pelagic *Sargassum* spp. seaweeds (*Sargassum natans* and *Sargassum fluitans*) were collected in the French West Indies (Saint-François beach, Guadeloupe), sun-dried, crushed and finally sieved to particle sizes ranging from 0.4 to 1 mm. The dried samples were then pyrolyzed in a tubular furnace (Thermolyne F 21100 (Dubuque, IA, USA)) under a nitrogen flow (80 ± 1 mL min^−1^) at temperatures varying from 600 to 900 °C. A heating rate of 10 °C min^−1^ was used, and the desired pyrolysis temperature was held for 3 h. The resulting black solid was thoroughly washed in a 5 M HCl solution for 12 h at 80 °C and then washed with water in a Soxhlet extractor to ensure that pH stabilization between 6 and 7 was reached [8]. Several carbons resulting from a pyrolysis temperature of 600, 700, 800 and 900 °C were obtained and will be later labelled CS600, CS700, CS800 and CS900, respectively.

### 3.2. Materials’ Characterization

The morphological features of samples were firstly investigated by scanning electron microscopy (SEM), using the Hitachi S-2500 (Ibaraki, Japan), combined with an energy-dispersive X-ray (EDX) microanalysis, to determine their chemical compositions. Analytical conditions varied as follows: secondary electrons mode (SE), electron beam voltage of 15 kV, work distance of 10.0–10.1 mm, spot size of 3.0–4.0 nm. The bulk elemental compositions were determined by taking EDX scans in different regions of the samples. Measurements were repeated 4–6 times and then averaged, leading to a relative error of ±10%. To simulate pyrolysis of the as-received Sargassum, thermogravimetric analysis (TGA) was carried out from 35 °C to 900 °C with a heating ramping rate of 10 °C min^−1^. The experiment was carried out using a thermogravimetric analyzer Labs Evo SETARAM (Kep Technologies, Caluire, France) with a precision of temperature of ±0.1 K and microbalance sensitivity of ±0.1 mg. For each experiment, 20 mg of sample was loaded into a platinum crucible and this was then introduced in the thermal analyzer. To maintain the inert condition, nitrogen was used as carrier gas with a flow rate of 80 ± 1 mL min^−1^. Each experiment was repeated at least twice for repeatability.

To evaluate the effect of pyrolysis on surface functional groups of algal biomass, Fourier transform infrared spectroscopy was also carried out in a Spectrum Two PerkinElmer (Villebon-sur-Yvette, France) spectrometer, with an attenuated total reflectance accessory (ATR/FT-IR). The spectra were recorded between 4000 and 550 cm^−1^ with a resolution of 8 cm^−1^. Triplicate scans of each sample were used to obtain an average spectrum, and the background spectrum was scanned under the same instrumental conditions. Surface properties of the CS samples were also analyzed by XPS (X-ray photoelectron spectroscopy). XPS measurements were conducted on an Axis-Ultra DLD Model from KRATOS, equipped with a hemispherical electron analyzer and a monochromatized Al-K (1486.6 eV) X-ray exciting source. The high sensitivity of the DLD detector with a source power of 90 W allowed the acquisition of high-quality spectra in a reasonable acquisition time. Identification and quantification of both elements and functional groups found on the surface of samples thus could be achieved. The instrument work function was calibrated to give a binding energy (BE) of 83.96 eV for the Au 4f7/2 line for metallic gold. The spectrometer dispersion was adjusted to give a BE of 932.62 eV for the Cu 2p3/2 line for metallic copper. No charge correction was applied to the spectra as the samples were conducting materials. The average relative error of the determined mass concentrations values was 10%. The Raman spectroscopy analysis was conducted on all samples, using a laser confocal. The excitation wavelength was 514 nm, with extended scans from 950 to 1800 cm^−1^. To determine the precise positions and intensity, the spectra were deconvoluted with Origin 8.5. 

Textural characterization for all samples was determined via nitrogen adsorption at 77 K, using an ASAP Micromeritics sorptiometer. Relative pressure (P/P_0_) ranged from 0.001 to 0.1. Prior to the analysis, the carbon samples were degassed for 24 h at 573 K to remove any moisture or adsorbed contaminants that may have been present on their surface. The specific surface area (S_BET_) of the carbons was calculated by applying the BET equation to adsorption data. The microporous surface (S_micro_), external surface (S_ext_), total pore volume (V_T_) and micropore volume (V_mi_) were evaluated by the t-plot method. The mesopore volume (V_me_) was estimated by the Barrett–Joyner–Halenda (BJH) method [33]. The mean pore diameter, D_p_, was calculated from D_p_ = 4V_T_/S assuming straight cylindrical pores, where V_T_ is the total volume of pores, and S the BET surface area. The average relative error of the determined parameters’ values was 10%.

For electrochemical studies, electrochemical cells were assembled in a two-electrode configuration using two carbon pellets as electrodes. The pellets were obtained by mixing 95% of the active material with 5% of the polytetrafluoroethylene (PTFE) binder at 60% in water, which was dispersed in ethanol [8]. After drying, the pellets were tested using a Teflon Swagelok cell construction, with a fiberglass separator soaked with an aqueous solution of sulfuric acid (0.5 M H_2_SO_4_). The mass loading and electrodes area were 15 mg cm^−2^ and 0.5 cm^2^, respectively.

Cyclic voltammetry (CV), galvanostatic charge/discharge and electrochemical impedance spectroscopy (EIS) were performed using a VMP multichannel potentiostat-galvanostat (Bio-logic, Seyssinet-Pariset, France). CV tests were performed between 0 and 1 V at rates of 1 and 2 mV s^−1^. Galvanostatic charge/discharge curves were measured in the potential range from 0 to 1 V, at a constant current density of 270 mA g^−1^. Impedance measurements were performed in the frequency range from 200 kHz to 10 mHz with a 5 mV voltage amplitude. The experiments were reproducible with an average error of ±5 F g^−1^ for capacitance values.

## 4. Conclusions

This study highlights that pelagic Sargassum, an invasive alga found in the Caribbean and the Gulf of Mexico, can serve as a cheap precursor for preparing carbon materials for electrochemical applications. By carefully controlling the synthesis conditions, such as the temperature of pyrolysis of the pelagic Sargassum, several mesoporous chars with large surface areas were successfully obtained. For instance, the carbon obtained from pyrolysis at 800 °C exhibited the highest microporous volume (0.92 cm^3^ g^−1^) and surface area (1664 m^2^ g^−1^), thus leading to the highest capacitance (96 F g^−1^) and the weakest resistance (0.073 Ω g^−1^), allowing the delivery of high electric power. These good electrochemical results seem to be linked to the textural characteristics of the biochars on the one hand, but also to the evolution of the structure on the other hand. Indeed, the O/C ratio decreases and the disorder within the biochars also decreases. This suggests a reorganization of the carbons within the materials.

The strong point of this work lies in the valorization of an algae considered as an important waste stranded on our coasts. By simple pyrolysis, carbon is generated with a large specific surface (1664 m^2^ g^−1^) and very interesting capacitive properties for the storage of electrical energy and capacitive deionization.

## Figures and Tables

**Table 1:** Characteristic FT-IR peaks for different functional groups of Sargassum.

Functional Groups	Position	Name	References
–OH, –NH	3660 cm^−1^	Alcohols, Amides	[13,16]
–CH_3_/–CH_2_	2960/2925 cm^−1^	Aliphatic hydrocarbons	[20]
–C=O	1700 cm^−1^	Aliphatic	[20]
–COOH/–COO^−^	1630 cm^−1^	Carboxyl acid/carboxylate	[13,16,20]
–C=C	1600 cm^−1^	Aromatic	[16,20]
–SO_3_	1416 cm^−1^	Sulfonate	[13,16]
–C–O–C	1230 cm^−1^	Ethers	[13,16]
–S=O	1320 cm^−1^	Sulfone	[16]
–S=O	1034 cm^−1^	Sulfonides	[16]
–C–H	700–900 cm^−1^	Aliphatic	[16,20]

**Table 2:** Elemental analysis and distribution of carbon and oxygen elements detected on the CS carbon surfaces from XPS data in %. Distribution of functional group detected on C1s spectrum; (I) graphite type, (II) carbonyl groups, (III) carboxyl and ester groups.

Elemental Composition (%)								Percentage of Functional Group from C1s Spectrum		
Sample	O_1s_	C_1s_	O/C	N_1s_	S_2p_	Si_2p_	Cl_2p_	(I)	(II)	(III)
CS 600	7.98	91.57	0.09			0.45		79.6	14.11	6.29
CS 700	10.19	87.16	0.12	0.71	0.75	1.19		79.1	15.45	5.45
CS 800	4.54	93.68	0.05		1.78			80.85	14.63	4.52
CS 900	2.76	96.33	0.03		0.59		0.32	82.43	13.28	4.29

**Table 3:** BET Surface area and porous texture parameters of CS samples.

Carbon	Specific Surface Area (m^2^ g^−1^)	Mesopore Volume (cm^3^ g^−1^)	Micropore Volume (cm^3^ g^−1^)	% of Micropore	Mean Pore Diameter (nm)
CS600	621	0.32	0.19	37.2	2.49
CS700	1179	0.51	0.39	43.3	2.32
CS800	1664	0.91	0.58	26.2	2.54
CS900	1199	0.60	0.39	39.4	2.44

**Table 4:** Whole cell capacitance from galvanometry measurements and other results from electrochemical impedance spectroscopy measurements (Pean model): equivalent series resistance ESR, in bulk electrolyte resistance *R*_Ω_, carbon electronic conductivity *Ri*, and *Rp*/3 effective in-pore ionic resistance.

Carbon	Csc whole Cell Capacitance (F g^−1^)	ESR (Ω g)	*R*_Ω_ in Bulk Electrolyte Resistance (Ω g)	*R_i_* (Carbon Electronic Conductivity) (Ω g)	*R_p_*/3 Effective In-Pore Ionic Resistance (Ω g)
CS _600_	89	0.33	0.0048	0.21	0.12
CS _700_	86	0.085	0.0041	0.065	0.016
CS _800_	96	0.073	0.0035	0.053	0.016
CS _900_	48	0.14	0.0035	0.12	0.013

**Table 5:** BET surface area, activation mode, and maximum capacitance of activated carbons from biomass precursors.

Precursor	Activation Method	S_BET_ (m^2^ g^−1^)	Capacitance (F g^−1^)	Electrolyte	Reference
Cassavapeelwaste	KOH	1352	153	0.5 M H_2_SO_4_	[28]
*Lessonia Nigrescens*	Pyrolysis 600 °C	746	264	1 M H_2_SO_4_	[7]
*Lessonia Nigrescens*	Pyrolysis 900 °C	1307	175	1 M H_2_SO_4_	[7]
Meristhoteca Senegalensis	Pyrolysis 750 °C	1156	120	1 M H_2_SO_4_	[7]
Lignin	NaOH/KOH		122	1 M H_2_SO_4_	[29]
Oil palm	H_2_O/CO_2_	1704	120	1 M H_2_SO_4_	[30]
Maple wood	Pyrolysis 750 °C	303	32	0.5 M H_2_SO_4_	[31]
Pineapple Leaves	Hydrothermal carbonization/KOH	1681	110	1 M H_2_SO_4_	[32]

## Data Availability

Data is unavailable due to privacy.
